# Complications after Chest Tube Removal and Reinterventions in Patients with Digital Drainage Systems

**DOI:** 10.3390/jcm8122092

**Published:** 2019-12-01

**Authors:** Yi-Ying Lee, Po-Kuei Hsu, Chien-Sheng Huang, Yu-Chung Wu, Han-Shui Hsu

**Affiliations:** Division of Thoracic Surgery, Department of Surgery, Taipei Veterans General Hospital, and School of Medicine, National Yang-Ming University, Taipei 100116, Taiwan; yylee10@vghtpe.gov.tw (Y.-Y.L.); huangcs@vghtpe.gov.tw (C.-S.H.); wuyc@vghtpe.gov.tw (Y.-C.W.); hsuhs@vghtpe.gov.tw (H.-S.H.)

**Keywords:** complication, digital drainage system, minimally invasive thoracic surgery

## Abstract

Introduction: Digital thoracic drainage systems are a new technology in minimally invasive thoracic surgery. However, the criteria for chest tube removal in digital thoracic drainage systems have never been evaluated. We aim to investigate the incidence and predictive factors of complications and reinterventions after drainage tube removal in patients with a digital drainage system. Method: Patients who received lung resection surgery and had their chest drainage tubes connected with a digital drainage system were retrospectively reviewed. Results: A total of 497 patients were monitored with digital drainage systems after lung resection surgery. A total of 175 (35.2%) patients had air leak-related complications after drainage tube removals, whereas 25 patients (5.0%) required reintervention. We identified that chest drainage duration of five days was an optimal cut-off value in predicting air leak-related complications and reinterventions. In multiple logistic regression analysis, previous chest surgery history; small size (16 Fr.) drainage tubes; the presence of initial air leaks, defined as air leaks recorded by the digital drainage system immediately after operation; and duration of chest drainage ≥5 days were independent factors of air leak-related complications, whereas the presence of initial air leaks and duration of chest drainage ≥5 days were independent predictive factors of reintervention after drainage tube removal. Conclusion: Air leak-related complications and reinterventions after drainage tube removals happened in 35.2% and 5.0% of patients with digital thoracic drainage systems. The management of chest drainage tubes in patients with predictive factors, i.e., the presence of initial air leaks and duration of chest drainage of more than five days, should be treated with caution.

## 1. Introduction

Postoperative air leaks are one of the most frequent complications after lung resection surgery. Since the introduction of the first digital thoracic drainage system in 2007 [1], its use has become increasingly popular in thoracic surgery. The features of digital thoracic drainage systems include the quantifying and recording of air leak flow rate and providing real-time continuous assessment of the severity of air leaks. Studies have demonstrated that the main advantages of digital thoracic drainage systems include early mobilization of the patients, objective evaluation of air leaks, reduced interobserver variability, and reduced duration of chest drainage, as well as hospital stay [2,3,4,5,6,7,8,9]. Moreover, it has been reported that digital drainage systems may enhance pleurodesis, leading to reduced recurrences after video-assisted thoracoscopic surgery (VATS) bullectomy and pleural abrasion [10].

However, the criteria for chest tube removal when using digital drainage systems are not conclusive. Whereas the ranges of air flow threshold vary from 0 to less than 40 mL/min for 6–12 h in studies, the optimal cut-off value and the reliability of these criteria have never been evaluated [2,3,4,5,6,7,8,9,10,11]. It has been reported that a significant portion of patients monitored with digital drainage systems still need chest tube reinsertion after chest tube removal [11]. However, the incidence of air-leak-related complications after chest drainage tube removal in patients with digital thoracic drainage systems and the predictive factors for the need for reinterventions remain unclear. The purpose of this paper is to review our experience using digital thoracic drainage systems in patients after lung resection surgery, with emphasis on patient outcome after drainage tube removal. Based on our observational data, we analyze the incidences of post-tube removal complications and factors associated with reinterventions, which would improve decision-making for chest drainage tube removals in patients with digital thoracic drainage systems.

## 2. Patients and Methods

This study was approved by the Institutional Review Board of the Taipei Veterans General Hospital and was granted a waiver for the requirement of informed consent (2017-01-016AC). We reviewed the data of patients who received thoracic surgery in the Taipei Veterans General Hospital and had their chest drainage tubes connected with a digital drainage system. The inclusion criteria were patients who received lung resection, including lobectomy, segmentectomy, and wedge resection. Patients with prior chest surgery history were also included, whereas those without lung resection (e.g., mediastinal tumor and esophageal cancer) or with incomplete clinical data (e.g., no preoperative pulmonary function tests or surgical mortality before drainage tube removal) were excluded.

The anesthesia methods during surgery included general anesthesia with either double lumen endotracheal tube insertions or single lumen endotracheal tubes with endobronchial blockers and nonintubated anesthesia without the use of endotracheal tubes. The surgical approaches included single-port VATS, multi-port VATS, and open thoracotomy. At the end of each procedure, an intraoperative underwater air leak test was performed routinely by submerging the lung parenchyma in sterile saline and reinflating the lung up to a sustained pressure of 20–30 cmH_2_O. Air bubble-producing defects were sealed using sutures repair, staples, sealants, or polyglycolic acid mesh. After closure of the chest wall wound, the pleural space was drained with either a pigtail (16 Fr.) or chest tube (20–28 Fr.), which were connected to the ThopazTM (Thopaz Digital Chest Drainage System, Medela AG, Baar, Switzerland) digital thoracic drainage system under continuous negative pressure of −5 to −20 cmH_2_O. The chest drainage tube was removed if the following criteria were satisfied: air leakage ≤ 20 mL/min for 4 consecutive h and a pleural fluid drainage amount less than 400 mL/24 h.

The primary outcome of this study was the incidence of air leak-related complications after drainage tube removal, including (1) subcutaneous emphysema, which is found either by physical examination or chest X-ray and (2) pneumothorax, which is defined as the presence of any air space on chest X-rays. The secondary outcome was the reintervention in patients monitored with digital drainage systems, including (1) the shift from digital drainage systems to traditional chest bottles and (2) reinsertion of chest drainage tubes due to the progression of subcutaneous emphysema and/or pneumothorax.

### Statistical Analysis

Categorical and continuous variables were compared with a Pearson’s chi-square test and the Wilcoxon rank-sum test. The independent predictors of the presence of air-related complications and reinterventions after drainage tube removal were determined using the multiple logistic regression model, which included significant factors in the univariate analysis. All statistical analyses were performed using Statistical Product and Service Solutions (version 25; IBM Corp, Armonk, New York, NY, USA), and a two-sided *p* value < 0.05 was considered significant.

## 3. Results

During the study period, 625 patients received chest drainage with a digital drainage system after thoracic surgery. Among them, 128 patients were excluded due to being without lung resection (*n* = 50), having incomplete preoperative pulmonary function tests (*n* = 77), and mortality before drainage tube removal (*n* = 1). A total of 497 patients were eligible and included in the analysis. 

The patient and surgical characteristics are shown in Table 1. A total of 175 (35.2%) of them had air leak-related complications after drainage tube removal, including progressive subcutaneous emphysema in 109 patients, pneumothorax in 81 patients, and both in 15 patients. The factors associated with air leak-related complications are shown in Table 1 and included male gender; smoking history, previous chest surgery history; poor forced expiratory volume in one second (FEV1); multi-port VATS or thoracotomy; lobectomy; larger size of drainage tubes; lower suction pressure (≤−10 cmH_2_O); presence of initial air leaks, which is defined as air leaks by digital thoracic drainage systems immediately after operation; longer duration of chest drainage; and diagnosis of primary lung cancer.

Among patients who met the primary outcome, 25 patients required reinterventions, including 23 patients who needed chest drainage tube reinsertion, one was shifted to a traditional drainage system on post-operation day 5, and one patient had both interventions. The factors associated with reinterventions included smoking history, lobectomy, lower suction pressure (≤−10 cmH_2_O), presence of initial air leaks, and longer duration of chest drainage. The predictors for reinterventions are shown in Table 2. 

Logistic regression analysis was used to identify predictive factors for air leak-related complications and reinterventions after drainage tube removals. In the multiple logistic regression analysis, previous chest surgery history, the use of a 16 Fr. pigtail catheter, the presence of initial air leaks, and the duration of chest drainage of more than five days were independent factors of post-drainage tube removal air leak-related complications (Table 3). Multiple logistic regression analysis also identified the presence of initial air leaks and the duration of chest drainage of more than five days as independent predictive factors for of reinterventions after drainage tube removals (Table 3).

## 4. Discussion

In recent years, digital drainage systems have become a useful adjunct to clinical management [11]. However, the benefit of digital drainage systems has been a debate in the literature, and trials have shown conflicting results [7,8,12,13,14,15]. In a prospective randomized study, the digital device protocol, in which chest tube removal was based on digitally recorded measurements, was compared to the traditional analog protocol, in which chest tube removal was based on an instantaneous assessment of air leak during daily rounds. It was concluded that via the objective data, it was possible to reduce the chest tube duration, hospital stay, and mean cost, by minimizing the interobserver variability in the decision-making process of removing chest tubes [7]. These results were reproduced in a multicenter international trial, which showed that patients managed with digital drainage systems had a shorter chest tube duration, shorter hospital stays, and significantly higher satisfaction scores when compared with those managed with traditional methods [8]. On the contrary, there were randomized trials showing that digital drainage systems did not reduce chest tube duration or length of hospitalization significantly compared with traditional water-sealed analog drainage systems [12,13,14,15]. Nevertheless, Gilbert and colleagues showed that the use of digital drainage systems significantly reduced the number of clamping attempts before the removal of the last remaining chest tube, indicating that the objective air leak trend data by the technology may serve as a more sensitive and reliable tool and may be clinically useful information [15]. Similarly, Plourde and colleagues demonstrated that digital devices provided the clinician with a more objective assessment of resolution of an air leak, which in turn, decreased the number of clamping trails and potentially the use of chest roentgenograms. However, this did not translate into a reduced chest tube duration or hospital length of stay in their study [12]. Moreover, they observed that the chest tube reinsertion rates were 8.4% and 5.6% in the digital group and analog group, respectively. They attributed the higher number of chest tube reinsertions in the digital group to the use of 30 mL/min as criteria for air leak cessations and concluded that the ideal rate of flow before chest tube removal is not clear [12]. Indeed, as high as 8% of patients managed with a digital system required drain reinsertion due to pneumothorax or subcutaneous emphysema after chest tube removal in the literature [12,16,17]. These findings inspired us to evaluate the reliability of data interpretation during the use of digital drainage systems. 

In this study, we focused on the incidence of air leak-related complications after chest drainage tube removals and the predictive factors for the need for reinterventions in patients with digital drainage systems. We defined pneumothorax and subcutaneous emphysema as air leak-related complications after chest drainage tube removal. Both are delayed presentations of a small amount of air leakage after chest drainage tube removal [18,19]. Although these may be subclinical in most cases, reinsertion of chest tubes may be needed once there is a certain amount of air leakage that the drainage system is unable to detect before tube removal. In our study, both complications were determined by strict criteria, which explained the high incidence (35.2%). We identified several factors that are associated with air leak-related complications, including patient factors such as positive smoking history and poor pulmonary function, which may be associated with poor wound healing, poor pleural symphysis, and compromised lung re-expansion [20,21]. In addition, previous chest surgery could be related to adhesion within the pleural cavity and higher possibility of parenchymal tear during pneumolysis, which may contribute to the prolonged duration of air leaks. In our study, multi-port VATS had higher complication rates than single-port VATS, which may be related to the conversion from single-port to multi-port VATS or even thoracotomy due to severe adhesion during surgery. Lobectomy was also associated with higher risks of both air leak-related complications and reinterventions compared with sublobar resections. We also found higher complication rates in chest tube groups compared with pigtail groups and lower suction pressure. The possible reason may be selective bias, because patients suspected of having a higher possibility of a longer duration of drainage tube according to intraoperative findings would receive chest tube drainage instead of a pigtail catheter, and surgeons in our department tend to use lower suction pressure in patients with more air leaks. Lastly, we found that initial air leaks were associated with both complication and reintervention rates. Therefore, careful dissection, early awareness of air leaks, and prevention strategies to decrease air leaks by all means, e.g., sealant use, in the operating room are important intraoperative factors that can reduce air leak-related complications [22]. 

With regard to reintervention rates in patients monitored with digital drainage systems, we also included patients shifting from digital drainage systems to traditional chest bottles, in addition to reinsertion of chest drainage tubes due to progression of complications. Because switching the drainage system usually means that clinicians are uncertain of the data displayed and the interpretation of the system, we defined it as an unreliable situation. In the literature, the chest tube reinsertion rate ranges from 0% to 8.4% [14,15,16,17]. In one recent study, 7.5% of patients using digital drainage systems needed reinsertion of chest drains, but reinsertion was significantly more frequent in the low-suction (−5 cmH2O, 13.2%) compared with the high-suction (−10 cmH2O, 1.9%) group [16]. In contrast, 7.2% required chest drain reinsertion due to pneumothorax or subcutaneous emphysema in another study, and there were no differences in the proportion or the size of the pneumothorax or subcutaneous emphysema after drain removal between suction of −2 cmH2O and −10 cmH2O [17]. In our study, the reintervention rate was 5.0%. The factors that were associated with reinterventions were smoking history, lobectomy, lower suction pressure (≤−10 cmH2O), presence of initial air leaks, and longer duration of chest drainage. We also identified chest drainage tubes present for more than five days as a critical factor for reintervention. Indeed, patients with a longer duration of intercostal drainage tube insertions represented a continuous or higher number air leaks. Based on our clinical experience, it is mandatory to examine the function of the chest drainage tube and the digital drainage system frequently for those with a drain duration of more than five days. Even if the criteria for drain removal have been met, it is important to rule out the possibility of drainage tube obstruction and drainage system dysfunction. If the amount of air leak flow on the system is not compatible with a patient’s clinical condition, chest roentgenogram or clamping trials are still needed for decision-making.

This study may have potential limitations due to its retrospective nature. For example, the use of sealants during operation, choice of drainage tubes, level of negative suction pressure of chest drainage tubes, and the criteria for reinsertion of drainage tubes were not standard among surgeons. The patient and surgical factors, e.g., the degree of pleural adhesion, the degree of fissure completeness, and the extent of resection, were heterogeneous in our study, which may be risks for air leak-related complications. All of these may have possibly influenced our results. Moreover, the cost effectiveness of digital drainage systems was not evaluated in this study.

In conclusion, we identified two factors, presence of initial air leaks and duration of chest drainage of more than five days, which were significantly predictive of air leak-related complications and reinterventions after chest drainage tube removals in patients monitored with digital drainage systems. Clinicians should be cautious when removing chest drainage tubes even when the criteria, which are based on the digital drainage system, are met. In the future, more studies are necessary to evaluate the outcome after chest drain removals and the reliability of digital drainage systems. 

## Figures and Tables

**Table 1:** Patient characteristics and factors associated with air leak-related complications after drainage tube removal.

Number (%)	Total (*n* = 497)	Air Leak-Related Complications (*n* = 175)	No Air Leak-Related Complications (*n* = 322)	*p* Value
Age (years, mean ± SD)	59.07 ± 15.13	60.81 ± 13.96	58.12 ± 15.67	0.050
Gender				0.012
Male	232 (46.7)	95 (54.3)	137 (42.4)	
Female	265 (53.3)	80 (45.7)	185 (57.5)	
BMI (Kg/m^2^, mean ± SD)	23.71 ± 3.29	23.74 ± 3.50	23.70 ± 3.18	0.831
Positive smoking history	127 (25.6)	65 (37.1)	62 (19.3)	<0.001
Previous chest surgery history	74 (14.9)	34 (19.4)	40 (12.4)	0.036
FEV1 (%, mean ± SD)	94.36 ± 18.12	91.63 ± 19.83	95.84 ± 16.97	0.008
Normal FEV1 (≥80%)	401 (80.7)	132 (75.4)	269 (83.5)	0.029
DLCO (%, mean ± SD)	68.03 ± 15.71	67.36 ± 15.11	68.39 ± 16.04	0.559
Normal DLCO (≥80%)	115 (23.1)	37 (21.1)	78 (24.2)	0.437
Surgical approach				<0.001
Single port VATS	286 (57.5)	79 (45.1)	207 (64.3)	
Multi-port VATS	197 (39.6)	90 (51.4)	107 (33.2)	
Open thoracotomy	14 (2.8)	6 (3.4)	8 (2.5)	
Lesion location *				
RUL	184 (37.0)	71 (40.6)	113 (35.1)	0.227
RML	85 (17.1)	27 (15.4)	58 (18.0)	0.465
RLL	140 (28.2)	56 (32.0)	84 (26.1)	0.162
LUL	141 (28.4)	49 (28.0)	92 (28.6)	0.893
LLL	103 (20.7)	33 (18.9)	70 (21.7)	0.449
Extent of resection				<0.001
Wedge resection	233 (46.9)	68 (38.9)	165 (51.2)	
Segmentectomy	63 (12.7)	15 (8.6)	48 (14.9)	
Lobectomy	200 (40.2)	92 (52.6)	108 (33.5)	
Bilobectomy	1 (0.2)	0	1 (0.3)	
Presence of pleural adhesion	51 (10.3)	20 (11.4)	31 (9.6)	0.527
Size of drainage tube (Fr.)				<0.001
16	211 (42.5)	42 (24)	169 (52.5)	
20	99 (19.9)	39 (22.3)	60 (18.6)	
24	86 (17.3)	39 (22.3)	47 (14.6)	
28	101 (20.3)	55 (31.4)	46 (14.3)	
Suction pressure (cmH_2_O)				0.001
≤−10	68 (13.7)	36 (20.6)	32 (9.9)	
>−10	429 (86.3)	139 (79.4)	290 (90.1)	
Presence of initial air leak	151 (30.4)	77 (44)	74 (23)	<0.001
Duration of chest drainage (d, mean ± SD)	4.9 ± 4.4	6.55 ± 5.6	4.05 ± 3.2	<0.001
Fluid amount on removal day (mL, mean ± SD)	101.6 ± 91.6	109.5 ± 93.3	97.3 ± 90.5	0.075
Diagnosis				0.020
Primary lung cancer	334 (67.2)	131 (74.9)	203 (63)	
Metastatic tumor	98 (19.7)	24 (13.7)	74 (23)	
Benign lesion	65 (13.1)	20 (11.4)	45 (14)	

SD: standard deviation; BMI: body mass index; FEV1: forced expiratory volume in one second; DLCO: Diffusing capacity of the lung for carbon monoxide; VATS: video-assisted thoracoscopic surgery; RUL: right upper lobe; RML: right middle lobe; RLL: right lower lobe; LUL: left upper lobe; LLL: left lower lobe. * Patients may have lesions over more than one lobe, and the *p* value indicates the significance level of whether a certain lobe contained the resected lesion.

**Table 2:** Factors associated with reinterventions after drainage tube removal.

Number (%)	Reintervention (*n* = 25)	No Reintervention (*n* = 472)	*p* Value
Age (years, mean ± SD)	64.2 ± 11.26	58.80 ± 15.27	0.057
Gender			0.075
Male	16 (64.0)	216 (45.8)	
Female	9 (36.0)	256 (54.2)	
BMI (Kg/m^2^, mean ± SD)	22.57 ± 2.92	23.77 ± 3.30	0.118
Positive smoking history	12 (48)	115 (24.4)	0.008
Previous chest surgery history	3 (12)	71 (15)	1.000
FEV1 (%, mean ± SD)	88.04 ± 22.54	94.69 ± 17.82	0.146
Normal FEV1 (≥80%)	17 (68.0)	384 (81.4)	0.117
DLCO (%, mean ± SD)	65.44 ± 17.27	68.17 ± 15.63	0.398
Normal DLCO (≥80%)	6 (24)	109 (23.1)	0.566
Surgical approach			0.510
Single port VATS	13 (52)	273 (57.8)	
Multi-port VATS	12 (48)	185 (39.2)	
Open thoracotomy	0	14 (3)	
Lesion location *			
RUL	11 (44.0)	173 (36.7)	0.458
RML	7 (28.0)	78 (16.5)	0.168
RLL	10 (40.0)	130 (27.5)	0.177
LUL	5 (20.0)	136 (29)	0.341
LLL	4 (16.0)	99 (21.0)	0.550
Extent of resection			0.027
Wedge resection	5 (20.0)	228 (48.3)	
Segmentectomy	3 (12.0)	60 (12.7)	
Lobectomy	17 (68.0)	183 (38.8)	
Bilobectomy	0	1 (0.2)	
Presence of pleural adhesion	2 (8.0)	49 (10.4)	1.000
Suction pressure (cmH_2_O)			0.013
≤−10	8 (32)	60 (12.7)	
>−10	17 (68)	412 (87.3)	
Size of drainage tube (Fr.)			0.220
16	6 (24.0)	205 (43.4)	
20	6 (24.0)	93 (19.7)	
24	6 (24.0)	80 (16.9)	
28	7 (28.0)	94 (19.9)	
Presence of initial air leak	18 (72.0)	133 (28.2)	<0.001
Duration of chest drainage (d, mean ± SD)	12.16 ± 9.52	4.54 ± 3.58	<0.001
Fluid amount on removal day (mL, mean ± SD)	115.84 ± 91.34	100.82 ± 91.65	0.439
Diagnosis			0.098
Primary lung cancer	21 (84.0)	313 (66.3)	
Metastatic tumor	1 (4.0)	97 (20.6)	
Benign lesion	3 (12.0)	62 (13.1)	

SD: standard deviation; BMI: body mass index; FEV1: forced expiratory volume in one second; DLCO: Diffusing capacity of the lung for carbon monoxide; VATS: video-assisted thoracoscopic surgery; RUL: right upper lobe; RML: right middle lobe; RLL: right lower lobe; LUL: left upper lobe; LLL: left lower lobe. * Patients may have lesions over more than one lobe, and the *p* value indicates the significance level in whether a certain lobe contained the resected lesion.

**Table 3:** Predictive factors of air leak-related complications and reinterventions after pleural drainage tube removal based on multiple logistic regression.

Variables	Odds Ratio (95% Confidence Interval)	*p* Value
Air leak-related complications after pleural drainage tube removal		
Previous chest surgery history	2.031 (1.144–3.605)	0.016
Size of drainage tube (16 Fr.)	0.483 (0.270–0.865)	0.014
Presence of initial air leaks	1.695 (1.088–2.641)	0.020
Chest drainage more than five days	2.178 (1.319–3.596)	0.002
Reinterventions after pleural drainage tube removal		
Presence of initial air leaks	4.342 (1.714–11.001)	0.002
Chest drainage more than five days	2.991 (1.005–8.905)	0.049
